# Prognostic Value of Complement Component 2 and Its Correlation with Immune Infiltrates in Hepatocellular Carcinoma

**DOI:** 10.1155/2020/3765937

**Published:** 2020-06-14

**Authors:** Gang Ning, Yan-Lin Huang, Li-Min Zhen, Wen-Xiong Xu, Xue-Jun Li, Li-Na Wu, Ying Liu, Chan Xie, Liang Peng

**Affiliations:** Department of Infectious Diseases, The Third Affiliated Hospital of Sun Yat-sen University, Guangzhou, China

## Abstract

**Background:**

Single nucleotide polymorphism (SNP) of complement component 2 (C2) has been found to be significantly associated with hepatocellular carcinoma (HCC). However, little is known about the role and mechanism of C2 in HCC. In the present study, we aimed to explore the prognostic value of C2 and its correlation with tumor-infiltrating immune cells in HCC.

**Materials and Methods:**

mRNA expression was downloaded from TCGA (365 HCC patients and 50 healthy controls), GSE14520 (220 HCC patients and 220 adjacent normal tissues), and ICGC HCC (232 HCC patients) cohorts. Unpaired Student's *t*-tests or ANOVA tests were used to evaluate differences of C2 expression. Univariate and multivariate analyses were used to analyze the prognostic value of C2. CIBERSORT was used to calculate the proportion of 22 kinds of tumor-infiltrating immune cells.

**Results:**

Significantly lower C2 expression was found at HCC compared to healthy controls, and C2 was associated with TNM stages. Higher C2 expression was significantly associated with better prognosis, and multivariate analysis showed that C2 was also an independent factor for the prognosis of HCC. Moreover, elevated CD4 T cells were found at HCC patients with higher C2 expression while the higher proportion of macrophage M0 cells was found in HCC patients with lower C2 expression. KEGG analysis showed that “cell cycle,” “AMPK signaling pathway,” and “PPAR signaling pathway” were enriched in HCC patients with higher C2 expression.

**Conclusion:**

C2 is a prognostic factor for HCC and may be used as a therapeutic target for future treatment of HCC.

## 1. Introduction

As one of the most common cancers and a leading cause of cancer-related death worldwide, hepatocellular carcinoma (HCC) continues to be a tremendous public burden on society [1]. Up to 50% of HCC patients are estimated to develop recurrence after resection partly due to limited amenable curative treatment options and rapid development of resistance [2]. A growing number of studies have indicated that tumor microenvironment (TME) plays important roles in almost every key aspect of HCC tumorigenesis, such as tumor initiation, progression, immune invasion, metastasis, recurrence, and resistance to therapy [3–5]. Therefore, understanding the interactions of stromal cells with cancer cells will help to develop effective strategies to conquer resistance and improve the therapeutic effect for HCC [6].

The complement system is a fundamental branch of innate immunity and could rapidly respond to invading pathogens by promoting cell lysis [7]. Remarkably, studies carried out over the last decade have shed new light on complement activation in the TME, which contributes to tumor-promoting and tumor-suppressing immune responses [8]. For example, complement component 7 (C7) and complement factor H (CFH) are found to be necessary for maintaining stemness of HCC cells as silence of C7 and CFH inhibits tumor-sphere formation and promotes cell differentiation while overexpression of them elevates stemness factor expression and cell growth in vivo [9]. C3 is required for the local and systemic immune responses against tumor in mice with G422 gliomas generated by photodynamic therapy (PDT), because knockout of C3 reduces the infiltration of immune cells and release of TNF-*α* and IFN-*γ*, which indicates a crucial role played by C3 in mediating antitumor immunity [10]. Interestingly, bilateral effect of complement component 5a (C5a) on tumor progression has also been observed. High levels of C5a are related to tumorigenesis accompanied by reduced IFN-*γ*-producing CD8 and CD4 cells, while a low level of C5a is associated with decreased tumor burden with increased IFN-*γ*-producing CD4 and CD8 T cells in mice with lymphoma [11]. These studies indicate entirely different roles played by each complement component on cancer development.

Complement component 2 (C2) is an important part of the complement system, and single nucleotide polymorphism (SNP) of C2 has been found to be significantly associated with HCC [12, 13]. For example, C2 SNP rs9267665 is associated with the risk of developing HCC while rs10947223 shows protective effects against HCC, indicating an important role played by C2 in HCC. However, up to now, little is known about the role and mechanism of C2 in HCC, so in the present study, we aimed to explore the prognostic value of C2 and its correlation with tumor-infiltrating immune cells of TME in HCC patients.

## 2. Materials and Methods

### 2.1. Ethics Statement

All the data analyzed in the present study were attained from The Cancer Genome Atlas (TCGA) dataset, Gene Expression Omnibus (GEO) dataset, and the International Cancer Genome Consortium (ICGC) dataset, and informed consents had been gained from each patient before our study.

### 2.2. Acquisition of mRNA Expression and Corresponding Clinical-Pathological Parameters from TCGA, GEO, and ICGC

In the present study, a total of three cohorts, including the TCGA HCC cohort, GSE14520 HCC cohort, and ICGC HCC cohort, were employed. mRNA expression and corresponding clinical-pathological parameters of the TCGA HCC cohort were downloaded from TCGA (https://cancergenome.nih.gov/). In the TCGA HCC cohort, clinical-pathological parameters of 377 HCC patients, including gender, age, histologic grades, cirrhosis, TNM stage, status, and time of overall survival (OS), were attained. Meanwhile, mRNA expression of 374 HCC patients and 50 healthy controls was also downloaded. The GSE14520 HCC cohort was downloaded from GEO (https://www.ncbi.nlm.nih.gov/geo/). In the GSE14520 HCC cohort, clinical-pathological parameters of 220 HCC patients, including gender, age, tumor size, cirrhosis, TNM staging, status, and time of OS, and mRNA expression of 220 HCC patients and their corresponding adjacent normal tissues were available. mRNA expression and corresponding clinical-pathological parameters of the ICGC HCC cohort were got from the ICGC portal (https://dcc.icgc.org/projects/LIRI-JP). In the ICGC HCC cohort, mRNA expression and clinical-pathological parameters of 232 HCC patients, including gender, age, TNM staging, status, and time of OS, were available. Basic demographic characteristics of TCGA, GSE14520, and ICGC HCC cohorts are summarized in Table 1.

### 2.3. CIBORSORT

An online tool, CIBERSORT, was used to calculate the proportion of 22 kinds of tumor-infiltrating immune cells with transcriptomic data (https://cibersort.stanford.edu). In CIBERSORT, relative fractions of 22 kinds of immune cells were deconvolved from the transcriptional expression of tumor samples basing on a referenced signature matrix by linear support vector regression.

### 2.4. Kyoto Encyclopedia of Genes and Genomes (KEGG) Analysis

The underlying mechanism of C2 in hepatocarcinogenesis was analyzed by KEGG analysis in the Database for Annotation, Visualization, and Integrated Discovery (DAVID) (https://david.ncifcrf.gov/summary.jsp). First, HCC patients of TCGA or GSE14520 or ICGC HCC cohort were divided into high C2 and low C2 groups according to the median C2 expression. Then, differentially expressed genes (DEGs) between the two groups were found with a cut-off value of *p* < 0.05. Finally, KEGG pathways enriched by these DEGs were identified in DAVID, and a cut-off value of *p* < 0.05 was considered as statistically significant.

### 2.5. Statistical Analysis

GraphPad Prism 6 (GraphPad Software, La Jolla, CA, USA) was used to carry out the statistical analyses. Data were presented as the median. Unpaired Student's *t*-tests or ANOVA tests were performed to compare the difference of C2 expression between HCC patients and healthy controls/adjacent normal tissues or among HCC patients with different histologic grades or TNM stages. Univariate and multivariate Cox regression analyses were carried out to analyze the prognostic value of C2 expression, and Kaplan-Meier analysis with a two-sided log-rank test was also performed to compare the OS of HCC patients with high or low C2 expression. Additional statistical analysis was performed with STAMP [14]. *p* < 0.05 was considered as statistically significant.

## 3. Results

### 3.1. C2 Expression between HCC Patients and Healthy Controls/Adjacent Normal Tissues

**Figure 1:**
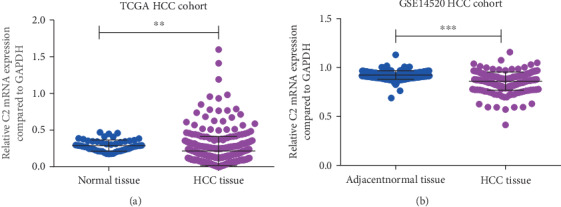
C2 expression between HCC patients and healthy controls/adjacent normal tissues. Expression of C2 in HCC patients and healthy controls of the TCGA cohort (a); expression of C2 in HCC patients and adjacent normal tissues of the GSE14520 cohort (b).

We first analyzed the C2 expression between HCC patients and healthy controls/adjacent normal tissues of TCGA and GSE14520 HCC cohorts. As is shown in Figure 1, in the TCGA cohort, significantly lower C2 expression was found at HCC patients compared to healthy controls (*p* < 0.01, Figure 1(a)). Similar results were also found in the GSE14520 cohort, in which significantly lower C2 expression was also found at HCC tissues compared to adjacent normal tissues (*p* < 0.001, Figure 1(b)). In short, the above results suggested that C2 expression of HCC was lower than that of healthy controls.

### 3.2. Association of C2 Expression with Clinical-Pathological Parameters of HCC Patients

**Figure 2:**
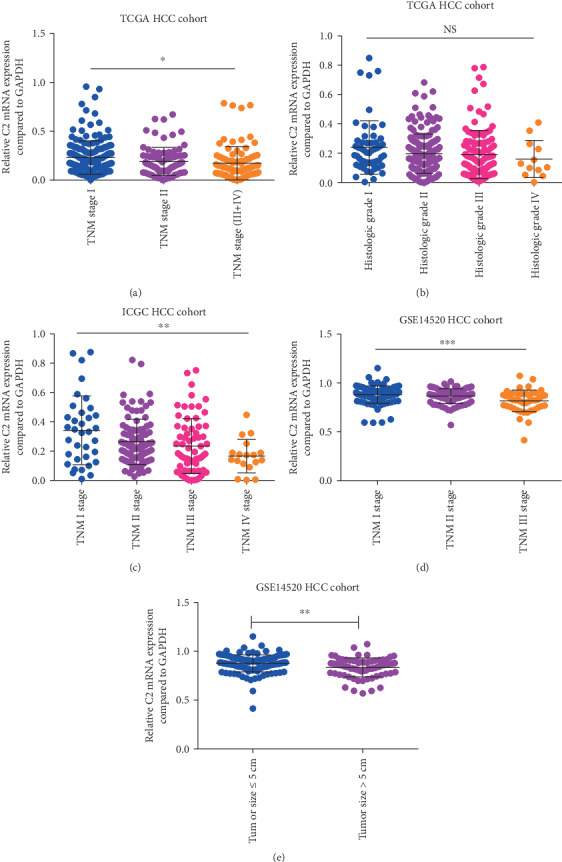
Association of C2 expression of HCC patients with clinical-pathological parameters. Association of C2 expression with TNM stages and histologic grades of TCGA cohort (a, b); association of C2 expression with TNM stages of ICGC cohort (c); association of C2 expression with TNM stages and main tumor size of cohort (d, e).

After reduced C2 expression was found in HCC patients, we next analyzed the association of C2 expression with clinical-pathological parameters. As is shown in Figure 2, in the TCGA cohort, C2 expression was remarkably correlated with TNM stages. HCC patients who were in more advanced TNM stages expressed lower C2 expression (*p* = 0.02, Figure 2(a)). Besides, HCC patients who were in more decreased differentiation tended to express lower C2 expression too, but the difference was not significant (*p* = 0.18, Figure 2(b)). Similar results were also found at ICGC and GSE14520 HCC cohorts. In the ICGC cohort, HCC patients who were in more advanced TNM stages expressed lower C2 expression (*p* = 0.004, Figure 2(c)). In the GSE14520 cohort, HCC patients who were in more advanced TNM stages expressed lower C2 expression (*p* < 0.0001, Figure 2(d)). Moreover, C2 expression of HCC patients with tumor size ≥ 5 cm was lower than that of HCC patients with tumor size ≤ 5 cm (*p* = 0.002, Figure 2(e)). Taken together, C2 expression was significantly associated with TNM stages in HCC patients, and the higher the TNM stages, the lower the C2 expression.

### 3.3. Prognostic Value of C2 in HCC Patients

**Figure 3:**
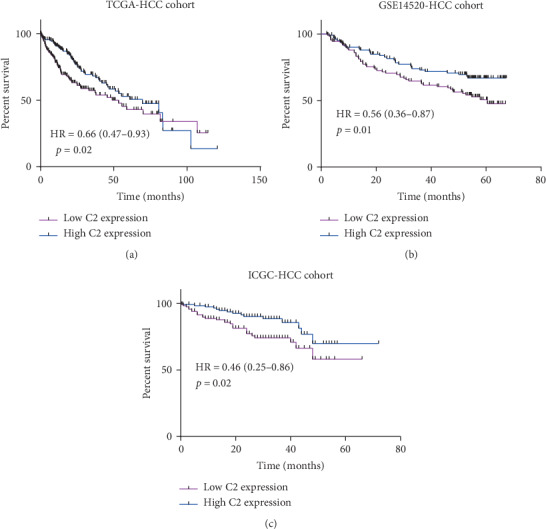
Prognostic value of C2 in HCC patients. Kaplan-Meier analysis of overall survival time of HCC patients with high C2 and low C2 expression in the TCGA cohort (a); Kaplan-Meier analysis of overall survival time of HCC patients with high C2 and low C2 expression in the GSE14520 cohort (b); Kaplan-Meier analysis of overall survival time of HCC patients with high C2 and low C2 expression in the ICGC cohort (c).

Having found that C2 expression was significantly associated with clinical-pathological parameters in HCC patients, we next analyzed the prognostic value of C2 in HCC patients. As is shown in Figure 3, C2 expression was significantly associated with the prognosis of HCC patients. In the TCGA cohort, univariate Cox analysis showed that higher C2 expression was significantly associated with better OS of HCC patients (HR = 0.66, 95% CI: 0.47-0.93, *p* = 0.02, Figure 3(a)). Moreover, multivariate analysis also showed that C2 expression was independently associated with OS of HCC patients after adjusting for gender, age, cirrhosis, histologic grade, and TNM stage (HR = 0.68, 95% CI: 0.47-0.99, *p* = 0.04, Table 2). In the GSE14520 cohort, univariate Cox indicated that higher C2 expression was also significantly associated with better OS of HCC patients (HR = 0.56, 95% CI: 0.36-0.87, *p* = 0.01, Figure 3(b)), but multivariate analysis showed that C2 expression was not independently associated with OS of HCC patients after adjusting for gender, age, cirrhosis, main tumor size, and TNM stage (HR = 0.73, 95% CI: 0.47-1.16, *p* = 0.18, Table 3). In the ICGC cohort, univariate Cox suggested that higher C2 expression was significantly related with better OS (HR = 0.46, 95% CI: 0.25-0.86, *p* = 0.02, Figure 3(c)), and multivariate analysis also showed that C2 expression was independently associated with OS of HCC patients after adjusting for gender, age, and TNM stage (HR = 0.73, 95% CI: 0.27-0.96, *p* = 0.04, Table 4). In a word, C2 may play an important role in HCC suppression.

### 3.4. Correlation of C2 Expression with Tumor-Infiltrating Immune Cells in HCC Patients

**Figure 4:**
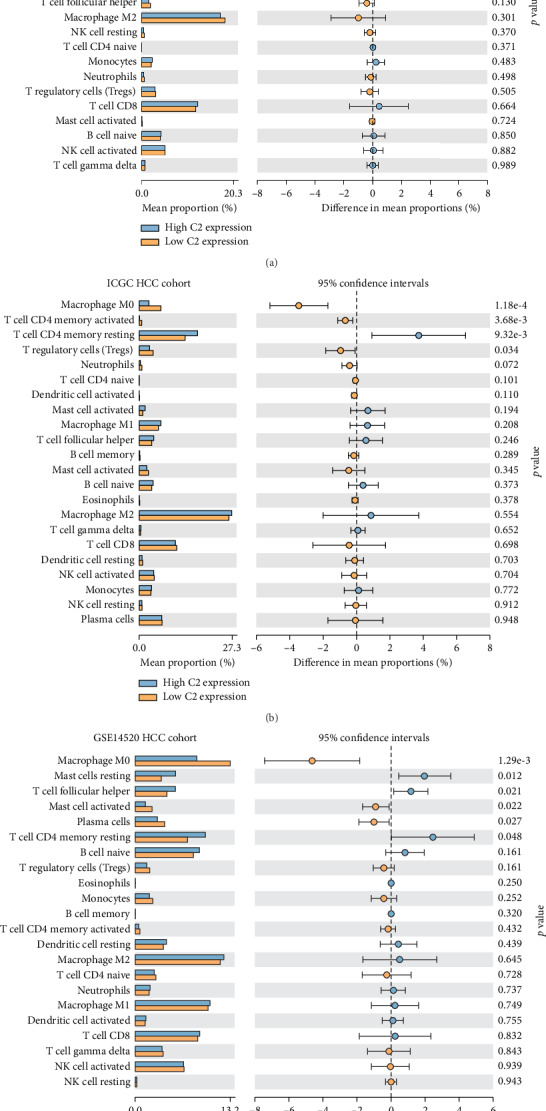
Correlation of C2 expression with tumor-infiltrating immune cells in HCC patients of the TCGA cohort (a), GSE14520 cohort (b), and ICGC cohort (c).

Increasing studies had proved an immunoregulatory effect of complement on TME, and interactions between complement and tumor-infiltrating immune cells contribute to the development and progression of many kinds of cancers [8]. So, we further analyzed the correlation of C2 expression with tumor-infiltrating immune cells in HCC patients. As is shown in Figure 4, C2 expression was significantly associated with tumor-infiltrating immune cells in HCC patients. In the TCGA cohort, significantly higher proportions of resting CD4 memory T cells and macrophage M1 cells were found in HCC patients with higher C2 expression, while significantly higher proportions of macrophage M0 cells, activated CD4 memory cells, and plasma cells were found at HCC patients with lower C2 expression (all *p* < 0.05, Figure 4(a)). In the GSE14520 cohort, significantly higher proportions of resting mast cells, follicular helper T cells, and resting CD4 memory T cells were found in HCC patients with higher C2 expression, while significantly higher proportions of macrophage M0 cells, activated mast cells, and plasma cells were found at HCC patients with lower C2 expression (all *p* < 0.05, Figure 4(b)). In the ICGC cohort, significantly higher proportions of resting CD4 memory T cells were found in HCC patients with higher C2 expression, while significantly higher proportions of macrophage M0 cells, activated CD4 memory T cells, and Treg cells were found at HCC patients with lower C2 expression (all *p* < 0.05, Figure 4(c)). Taken together, C2 expression was significantly associated with CD4 memory T cells and macrophage M0 cells in HCC patients from all these three HCC cohorts.

### 3.5. Molecular Mechanisms of C2 in HCC

**Figure 5:**
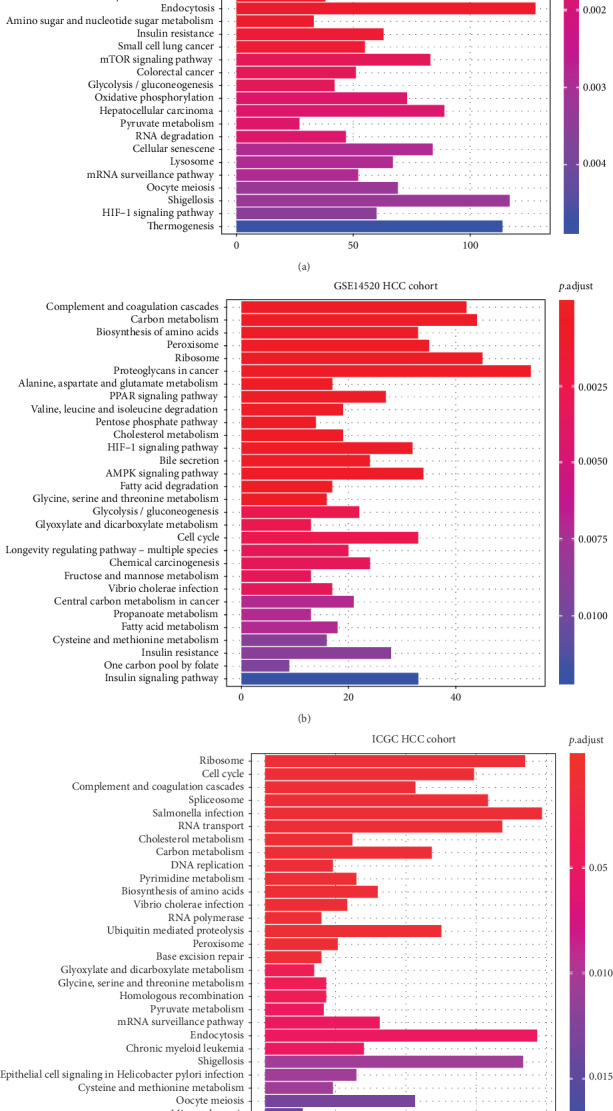
The most significant 30 KEGG pathways enriched in HCC patients with higher C2 expression compared to HCC patients with lower C2 expression of the TCGA cohort (a), GSE14520 cohort (b), and ICGC cohort (c).

KEGG analysis was performed to explore the underlying biological mechanism by which C2 influenced the prognosis of HCC. As is shown in Figure 5, in the TCGA cohort, KEGG pathways, such as “cell cycle,” “ubiquitin-mediated proteolysis,” “complement and coagulation cascades,” “spliceosome,” and “RNA transport,” were most significantly enriched in HCC patients with higher C2 expression compared to HCC patients with lower C2 expression (all *p* < 0.001, Figure 5(a)). In the GSE14520 cohort, KEGG pathways, such as “complement and coagulation cascades,” “carbon metabolism,” “biosynthesis of amino acids,” “peroxisome,” and “ribosome,” were most significantly enriched in HCC patients with higher C2 expression (all *p* < 0.001, Figure 5(b)). In the ICGC cohort, KEGG pathways, such as “ribosome,” “cell cycle,” “complement and coagulation cascades,” “spliceosome,” and “RNA transport,” were most significantly enriched in HCC patients with higher C2 expression (all *p* < 0.001, Figure 5(c)). In total, as is shown in Table 5, 17 significant KEGG pathways, such as “cell cycle,” “complement and coagulation cascades,” “AMPK signaling pathway,” and “PPAR signaling pathway,” overlapped in the three HCC cohorts, indicating that C2 may influence the prognosis of HCC by regulation of these 17 overlapped KEGG pathways.

## 4. Discussion

HCC is one of the most malignant kinds of cancer worldwide, and TME has been found to play important roles of recurrence and resistance to therapy of HCC [8, 15]. Increasing studies have proved an immunoregulatory effect of complement on TME, and interactions between complement and TME contribute to the development and progression of many kinds of cancers [8]. C2 is an important part of the complement system, and SNP of C2 has been found to be significantly associated with HCC [12, 13]. In the present study, we found that C2 was associated with the prognosis of HCC and explored the relevant underlying mechanism.

Previously, Imamura et al. have found that higher expression of the C5a receptor from breast cancer tissues is significantly associated with clinical parameters, such as larger tumor size, advanced histologic grade, lymph node metastasis, higher TNM stages, and poorer prognosis [16]. Lin et al. have also observed that higher tissue C3 expression is significantly associated with better prognosis of non-small-cell lung cancer (NSCLC) patients, indicating an important role played by C3 in NSCLC suppression [17]. Similarly, in our study, lower expression of C2 was found at HCC patients, and C2 expression was significantly associated with TNM stages and better OS. Besides, C2 expression was independently associated with OS of HCC patients in TCGA and ICGC HCC cohorts, but it was not in the GSE14520 cohort. The reason for the difference in the predictive value of C2 expression in different HCC cohorts may be that some pathological parameters among the three HCC cohorts were different. For example, in the TCGA HCC cohort, cirrhosis was not associated with OS, but in the GSE14520 HCC cohort, cirrhosis and main tumor size were significantly associated with OS. Moreover, in the TCGA and GSE1420 HCC cohorts, gender was not associated with OS, but in the ICGC HCC cohort, gender was associated with OS. The difference of cirrhosis, main tumor size, and gender among the three HCC cohorts may result in the difference of factors used for multivariate analysis, thus leading to the difference of the predictive value of C2 expression for OS. In a word, C2 expression was associated with OS of HCC patients, but it still needed external and multicenter prospective cohorts with large sample sizes to validate whether C2 expression could be an independent prognostic factor for OS.

A series of studies have proved that the complement system takes part in the regulation of TME, and interaction of complement with tumor-infiltrating immune cells plays an important role in the development and progression of many kinds of cancers [8]. Tumor cell-derived C5a can recruit and differentiate myeloid-derived suppressor cells (MDSCs) in TME, which could protect tumor cells against the immune system and immunotherapy and promote tumor progression by inhibiting T cell responses and promoting the generation of Tregs [18]. Moreover, blockade of C5aR can reduce the number of MDSCs [19]. Lin et al. have also showed that higher tissue C3 expression was positively correlated with higher numbers of tumor-infiltrating CD4 T cells and CD8 T cells, which may also contribute to tumor suppression by C3, as higher C3 expression predicts better prognosis in NSCLC patients [17]. In the present study, elevated CD4 T cells were found at HCC patients with higher C2 expression while a higher proportion of macrophage M0 cells was found in HCC patients with lower C2 expression in all three HCC cohorts. Garnelo et al. have showed that the degree of infiltrated T cells and B cells of tumor tissues significantly relates to the improved prognosis of HCC patients [20]. CD4+ T cells can improve antitumor immune responses by producing cytokines which are important for the activation of CD8+ T cells and B cells. Imai et al. have found that CD4+ T cells are important for the formation and maintenance of polyfunctionality of cytotoxic CD8+ T cells, which is a key determinant of the success of immunological control of tumor growth [21]. Moreover, the important role of virus control and antitumor immunity played by CD4+ T cell-mediated cytotoxicity is being increasingly recognized. Fu et al. have found that circulating and tumor-infiltrating CD4+ cytotoxic T cells decrease in HCC patients with advanced stages, and loss of CD4+ cytotoxic T cells is associated with a high mortality rate and reduced survival time [22]. Hsiao et al. have observed that the higher number of macrophage M0 cells is significantly associated with poorer prognosis of HCC patients [23]. Macrophages can be recruited into HCC tissue to become tumor-associated macrophages (TAMs) by upregulation of HMGB1 and then take part in the cancer progression and metastasis [24]. TAMs locate in the stroma of HCC tissue and are polarized toward the M2 phenotype. A lot of studies have showed that TAMs can promote tumor proliferation, angiogenesis, invasion, and metastasis [25]. For example, Yeung et al. have showed that M2 macrophages are associated with a poor prognosis in HCC by promoting tumor growth and invasiveness through CCL22-induced epithelial-mesenchymal transition (EMT) [26]. Besides, tumor-suppressive M1 macrophages were found to be enriched in HCC patients with higher C2 expression in the TCGA cohort, which may contribute to better prognosis of HCC patients as studies have showed that M1 macrophages are involved in killing pathogens and tumor cells by producing large amounts of proinflammatory cytokines and expressing MHC molecules [27]. Moreover, the higher proportion of Tregs was found at HCC patients with lower C2 expression in the ICGC cohort, which may also contribute to the unfavorable prognosis of HCC patients as Treg cells are immunosuppressive cells and could promote the occurrence and development of HCC by inhibiting the function of T cells [28]. Taken together, the interaction of C2 with tumor-infiltrating immune cells may influence the prognosis of HCC.

In addition, to analyze the association of C2 expression with tumor-infiltrating immune cells, we also explore the underlying signaling pathways exploited by C2 to influence prognosis by KEGG analysis. Signaling pathways, such as “AMPK signaling pathway” and “PPAR signaling pathway,” were found to be significantly enriched in HCC patients with higher C2 expression from all three HCC cohorts, which have been reported to take part in the development and progression of HCC. For example, Han et al. have observed that hispidulin could suppress the growth and metastasis of HCC through AMPK signaling-mediated PPAR*γ* activation both in vitro and in vivo [29]. Tuo et al. have also found that phosphoenolpyruvate carboxykinase 1 (PCK1), one of the key enzymes of gluconeogenesis, could inhibit the progression of cell cycle and proliferation of hepatoma cells via the AMPK/p27^Kip1^ axis [30]. Basing on these studies, we speculated that C2 may influence the prognosis of HCC by regulation of the AMPK signaling pathway and/or PPAR signaling pathway.

Several limitations of the present study should also be noted. First, complement is mainly synthesized in the liver and then secreted into blood, but we do not analyze the difference of serum C2 between HCC and healthy controls and the prognostic value of serum C2 in HCC. Second, C2 expression is found to be associated with tumor-infiltrating cells, such as CD4 T cells and macrophage M0 cells, but we do not explore how C2 regulate these immune cells to influence the prognosis of HCC patients. Finally, we do not validate the KEGG pathways enriched in HCC patients with higher C2 expression in in vitro studies.

In conclusion, higher C2 expression is associated with better prognosis of HCC, and C2 may influence the prognosis of HCC by interaction with CD4 T cells and macrophage M0 cells and regulation of pathways, such as the AMPK signaling pathway and PPAR signaling pathway.

## Figures and Tables

**Table 1:** Basic characteristics of HCC patients from the TCGA, GSE14520, and ICGC HCC cohorts.

Variables	TCGA cohort (*N* = 377)	GSE14520 cohort (*N* = 220)	ICGC cohort (*N* = 232)
Gender (male/female)	255/122	190/30	171/61
Age (years, median)	60 (16-90)	50 (21-77)	69 (31-89)
Cirrhosis (yes/no/NA)	81/137/159	202/18	NA
Histologic grade (G1/G2/G3/G4/NA)	55/180/124/13/5	NA	NA
TNM stage (I/II/III/IV/NA)	175/87/86/5/24	93/77/48/-/2	36/106/71/76
Main tumor size (>/≤5 cm)	NA	80/140	NA
Overall survival status (alive/dead)	245/132	136/84	189/43
Overall survival time (months, median)	19.67 (0-120.73)	51.65 (1.8-67.4)	26.0 (0.3-72)

**Table 2:** Univariate and multivariate analyses of C2 expression for overall survival in HCC patients of the TCGA cohort.

Variables	Univariate analysis			Multivariate analysis		
	Hazard ratio	95% CI	*p* value	Hazard ratio	95% CI	*p* value
Gender (male vs. female)	0.82	0.57-1.16	0.26			
Age (>60 vs. ≤60)	1.25	0.88-1.77	0.21			
Cirrhosis (yes vs. no)	0.83	0.48-1.42	0.49			
Histologic grade (G3+G4 vs. G1+G2)	1.12	0.78-1.61	0.54			
TNM stage (III+IV vs. I+II)	2.43	1.69-3.55	0.000^∗^	2.38	1.64-3.46	0.000^∗^
C2 (high vs. low)	0.66	0.47-0.93	0.02^∗^	0.68	0.47-0.99	0.04^∗^

**Table 3:** Univariate and multivariate analysis of C2 expression for overall survival in HCC patients of the GSE14520 cohort.

Variables	Univariate analysis			Multivariate analysis		
	Hazard ratio	95% CI	*p* value	Hazard ratio	95% CI	*p* value
Gender (male vs. female)	1.68	0.81-3.49	0.16			
Age (>50 vs. ≤50)	1.0	0.65-1.53	0.99			
Cirrhosis (yes vs. no)	4.57	1.13-18.62	0.03^∗^	3.66	0.89-14.98	0.07
Main tumor size (>5 cm vs. ≤5 cm)	1.97	1.28-3.30	0.002^∗^	1.18	0.69-2.01	0.56
TNM stage (III+IV vs. I+II)	3.43	2.20-6.11	0.000^∗^	2.38	1.64-3.46	0.000^∗^
C2 (high vs. low)	0.56	0.36-0.87	0.01^∗^	0.73	0.47-1.16	0.18

**Table 4:** Univariate and multivariate analyses of C2 expression for overall survival in HCC patients of the ICGC cohort.

Variables	Univariate analysis			Multivariate analysis		
	Hazard ratio	95% CI	*p* value	Hazard ratio	95% CI	*p* value
Gender (male vs. female)	0.51	0.28-0.97	0.04^∗^	0.43	0.23-0.81	0.01^∗^
Age (>70 vs. ≤70)	1.06	0.58-1.94	0.84			
TNM stage (III+IV vs. I+II)	2.38	1.30-4.36	0.004^∗^	2.59	1.39-4.80	0.003^∗^
C2 (high vs. low)	0.46	0.25-0.86	0.02^∗^	0.73	0.27-0.96	0.04^∗^

**Table 5:** Overlap of significant KEGG pathways enriched in HCC patients with higher C2 expression from the TCGA, GSE14520, and ICGC HCC cohorts.

ID	Description	TCGA cohort		GSE14520 cohort		ICGC cohort	
		Count	*p*.adjust	Count	*p*.adjust	Count	*p*.adjust
hsa04110	Cell cycle	96	0.000	33	0.003	89	0.000
hsa04610	Complement and coagulation cascades	59	0.000	42	0.000	64	0.000
hsa04152	AMPK signaling pathway	61	0.023	34	0.001	58	0.031
hsa03320	PPAR signaling pathway	42	0.014	27	0.000	40	0.025
hsa04910	Insulin signaling pathway	80	0.000	28	0.008	66	0.026
hsa01200	Carbon metabolism	73	0.000	44	0.000	71	0.000
hsa00630	Glyoxylate and dicarboxylate metabolism	20	0.012	13	0.002	21	0.004
hsa00620	Pyruvate metabolism	27	0.001	13	0.017	25	0.006
hsa01230	Biosynthesis of amino acids	42	0.011	33	0.000	48	0.000
hsa00410	Beta-alanine metabolism	19	0.039	11	0.021	19	0.030
hsa00270	Cysteine and methionine metabolism	29	0.017	16	0.008	29	0.012
hsa05110	Vibrio cholerae infection	31	0.006	17	0.004	35	0.000
hsa00010	Glycolysis/gluconeogenesis	42	0.001	22	0.002	36	0.029
hsa04979	Cholesterol metabolism	36	0.000	19	0.001	37	0.000
hsa00280	Valine, leucine, and isoleucine degradation	27	0.042	19	0.000	27	0.029
hsa00640	Propanoate metabolism	21	0.027	54	0.000	20	0.041
hsa00260	Glycine, serine, and threonine metabolism	23	0.048	16	0.001	26	0.004

## Data Availability

All the data used in the present study could be downloaded from TCGA (https://cancergenome.nih.gov/), GEO (https://www.ncbi.nlm.nih.gov/geo/), and ICGC portal (https://dcc.icgc.org/projects/LIRI-JP).

## Abbreviations

HCC: Hepatocellular carcinoma
TME: Tumor microenvironment
C7: Complement component 7
CFH: Complement factor H
PDT: Photodynamic therapy
C5a: Complement component 5a
C2: Complement component 2
SNP: Single nucleotide polymorphism
TCGA: The Cancer Genome Atlas
GEO: Gene Expression Omnibus
ICGC: International Cancer Genome Consortium
OS: Overall survival
KEGG: Kyoto Encyclopedia of Genes and Genomes
DAVID: Database for Annotation, Visualization, and Integrated Discovery
DEGs: Differentially expressed genes
NSCLC: Non-small-cell lung cancer
MDSCs: Myeloid-derived suppressor cells
EMT: Epithelial-mesenchymal transition
MBL: Mannan binding lectin
PCK1: Phosphoenolpyruvate carboxykinase 1.
